# Pharmacologic Treatment Assigned for Niemann Pick Type C1 Disease Partly Changes Behavioral Traits in Wild-Type Mice

**DOI:** 10.3390/ijms17111866

**Published:** 2016-11-09

**Authors:** Victoria Schlegel, Markus Thieme, Carsten Holzmann, Martin Witt, Ulrike Grittner, Arndt Rolfs, Andreas Wree

**Affiliations:** 1 Institute of Anatomy, University of Rostock, 18055 Rostock, Germany; victoria.schlegel@gmx.de (V.S.); markus_thieme@gmx.de (M.T.); martin.witt@med.uni-rostock.de (M.W.); 2 Institute of Medical Genetics, Rostock University Medical Center, 18057 Rostock, Germany; carsten.holzmann@med.uni-rostock.de; 3 Department for Biostatistics and Clinical Epidemiology, Charité—Universitätsmedizin Berlin, 10117 Berlin, Germany; ulrike.grittner@charite.de; 4 Albrecht-Kossel Institute for Neuroregeneration, Rostock University Medical Center, 18147 Rostock, Germany; arndt.rolfs@med.uni-rostock.de

**Keywords:** cyclodextrin, allopregnanolone, miglustat, NPC, mice, behavior, accelerod, Morris water maze, elevated plus maze, open field, hot-plate

## Abstract

Niemann-Pick Type C1 (NPC1) is an autosomal recessive inherited disorder characterized by accumulation of cholesterol and glycosphingolipids. Previously, we demonstrated that BALB/c-npc1^nih^*Npc1*^−/−^ mice treated with miglustat, cyclodextrin and allopregnanolone generally performed better than untreated *Npc1*^−/−^ animals. Unexpectedly, they also seemed to accomplish motor tests better than their sham-treated wild-type littermates. However, combination-treated mutant mice displayed worse cognition performance compared to sham-treated ones. To evaluate effects of these drugs in healthy BALB/c mice, we here analyzed pharmacologic effects on motor and cognitive behavior of wild-type mice. For combination treatment mice were injected with allopregnanolone/cyclodextrin weekly, starting at P7. Miglustat injections were performed daily from P10 till P23. Starting at P23, miglustat was embedded in the chow. Other mice were treated with miglustat only, or sham-treated. The battery of behavioral tests consisted of accelerod, Morris water maze, elevated plus maze, open field and hot-plate tests. Motor capabilities and spontaneous motor behavior were unaltered in both drug-treated groups. Miglustat-treated wild-type mice displayed impaired spatial learning compared to sham- and combination-treated mice. Both combination- and miglustat-treated mice showed enhanced anxiety in the elevated plus maze compared to sham-treated mice. Additionally, combination treatment as well as miglustat alone significantly reduced brain weight, whereas only combination treatment reduced body weight significantly. Our results suggest that allopregnanolone/cyclodextrin ameliorate most side effects of miglustat in wild-type mice.

## 1. Introduction

Niemann-Pick Type C1 (NPC1) is an autosomal recessive lysosomal storage disease caused by mutations in the *NPC1* gene located on chromosomal band 18q11 [1]. Lack of functional NPC1 protein leads to abnormal intracellular trafficking of cholesterol and results in accumulation of unesterified cholesterol in late endosomes/lysosomes [2].

The widely used BALB/c-npc1^nih^*Npc1*^−/−^ mouse model [3] partly mimics the human disease resulting in neuronal lipid storage and progressive neurodegeneration, particularly seen in a dramatic loss of Purkinje cells [4].

Therapeutic options for NPC1 disease are limited. Up to date, three drugs were reported to have positive effects on lifespan and clinical signs of *Npc1* mutant mice. Miglustat administration was shown to slow down the progression of the disease [5]. Miglustat, an inhibitor of glucosylceramide synthase, a key component of the glycosphingolipid biosynthetic pathway, reduced toxic metabolites like sphingomyelin, sphingosine, cerebrosides and other complex glycosphingolipids. By this, miglustat can improve clinical symptoms of NPC1 disease in humans [6,7,8,9] and is well tolerated in NPC1 patients after long-term application [10]. Thus, miglustat was approved for use in treatment of neurologic symptoms of Gaucher and NPC diseases by the European Medicines Agency.

However, administration of allopregnanolone and its solvent 2-hydroxypropyl-β-cyclodextrin (HPβCD), without miglustat, also increased the lifespan of *Npc1*^−/−^ mice, delayed the age of onset of symptoms and reduced ganglioside accumulation [11]. Recently, a combination therapy (cyclodextrin, allopregnanolone, miglustat) has been shown to delay disease onset, reduce intraneuronal lipid storage, reduce cerebellar neurodegeneration [12] and ameliorate motor but not cognitive deficits in *Npc1* mutant mice [13,14]. However, since treated mutant mice were compared to sham-treated mutant and wild-type mice no data exist about the behavioral effects of the drugs on healthy wild-type mice. Moreover, it seemed important to evaluate the consequences of therapeutically used drugs in wild-type animals in order to study specific negative effects or to show that no side effects occurred. Interestingly, HPβCD was shown to have no effect on visual evoked potential response in wild-type mice [15], whereas others reported severe ototoxicity of exactly this drug in mice and cats [16,17,18].

Using a battery of standard behavioral tests, we present the first study of pharmacologic effects of miglustat as singularly administered substance in comparison to the well-known combination therapy with β-cyclodextrin/allopregnanolone/miglustat [12] on motor, psychiatric and cognitive capabilities as well as pain sensitivity of wild-type mice. Moreover, we wanted to test for possible side effects that will become evident when drugs are chronically applied to healthy mice from P7 onwards.

## 2. Results

### 2.1. Reduced Body Weights after Combination Treatment

**Figure 1:**
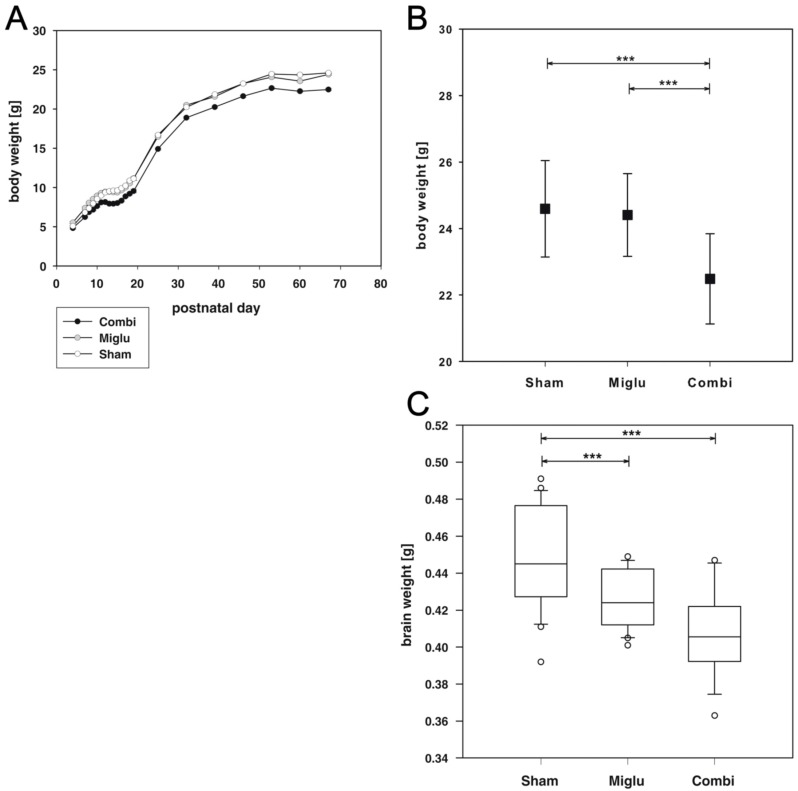
Weights. (**A**) Body weight values of postnatal day 4 to 67 mice. Sham-treated mice (*n* = 29) are displayed with open circles, miglu-treated mice (*n* = 26) with grey filled circles and combi-treated animals (*n* = 26) with black filled circles; (**B**) body weights at day of sacrifice; (**C**) brain weights at day of sacrifice. Scatter plot data are represented as mean ± SD. Box plots depict the groups graphically by displaying the following descriptive statistical parameters: the median, the upper and lower quartiles, and outliers (circles) that lie outside the 10th and 90th percentiles (whiskers). Significant post hoc effects are indicated by asterisks (*** *p* < 0.001).

The mice of all groups were weighted on all injection days, i.e., P7 to P63 and before sacrifice, i.e., P67 (Figure 1A). Two way repeated measures ANOVA revealed highly significant differences for treatment (F_2, 78_ = 22.402, *p* < 0.001). The body weights at day of sacrification are significantly different (F_2, 78_ = 19.643, *p* < 0.001). The mean body weight of sham-treated mice was 24.6 g (SD: 1.5 g), for combination-treated mice 22.5 g (SD: 1.6 g) and 24.4 g (SD: 1.3 g) for miglustat-treated mice. Post hoc all pairwise multiple comparison procedures revealed that the average body weight of the combi-group was significantly reduced compared to the sham-group and the miglu-group (both *p* < 0.001), whereas body weights of sham- and miglu-groups did not significantly vary (*p* = 0.610) (Figure 1B).

### 2.2. Reduced Brain Weights after Miglustat- and Combination Treatment

Analysis of brain weights using one way ANOVA on ranks revealed a statistically significant difference (H_2_ = 19.091, *p* < 0.001). All pairwise multiple comparison procedures (Dunn’s method) showed that the brains of combination- (mean: 0.408 g, SD: 0.0234 g) and miglustat-treated mice (mean: 0.426 g, SD: 0.0156 g) were significantly lighter (*p* < 0.001) compared to sham-treated mice (mean 0.449 g, SD: 0.0278 g, Figure 1C).

### 2.3. Miglustat and Combination Treatment Had No Effects on Motor Capabilities

**Figure 2:**
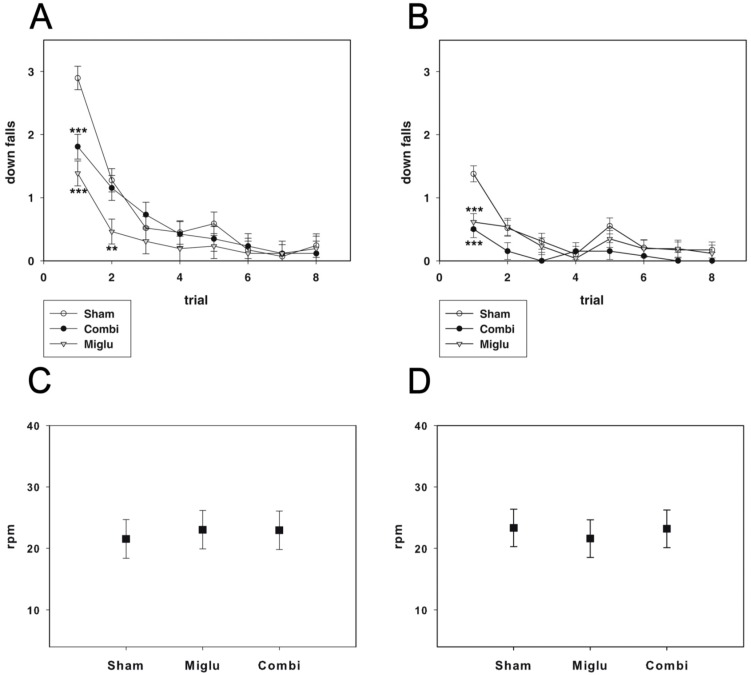
Accelerod test. (**A**,**B**) At P35 (**A**) and P60 (**B**) the animals of each group (*n*_sham_ = 29, *n*_miglu_ = 26, *n*_combi_ = 26) reached a constant level of down falls after 6 training runs; (**C**,**D**) in the probe trials of the accelerod test there were no significant differences for P35 (**C**) and P60 (**D**), respectively. Data are presented as mean ± SEM (**A**,**B**) or mean ± SD (**C**,**D**). Asterisks indicate significant differences compared to the sham group (** *p* < 0.01, *** *p* < 0.001).

For evaluating motor coordination and balance, the accelerod test was performed. Animals of all groups learned the task during both training trials, indicated by decreasing numbers of down falls during the course of the training (P35: F_14, 546_ = 2.944, *p* < 0.001; P60: F_14, 546_ = 2.223, *p* = 0.006). Sham-treated mice started worse than miglustat- and combination-treated mice. All pairwise multiple comparison procedures (Holm–Sidak method) revealed significant differences (*p* < 0.001) for training trials 1 and 2 at p35 and for training trial 1 at P60 (Figure 2A,B). However, during further training we did not detect any significant differences in motor performance between the three treatment groups.

During probe trials with accelerating speed of the treadmill we also detected no statistically significant difference between the treatment groups with regard to reached speed at down fall at P35 and P60 (P35: F_2, 79_ = 2.012, *p* = 0.141; P60: F_2, 79_ = 2.684, *p* = 0.074) (Figure 2C,D). In conclusion, neither miglustat nor combination treatment caused alteration of motor coordination and balance in comparison with sham-treated mice.

### 2.4. Impaired Spatial Learning by Miglustat Treatment but Not by Combination Treatment

**Figure 3:**
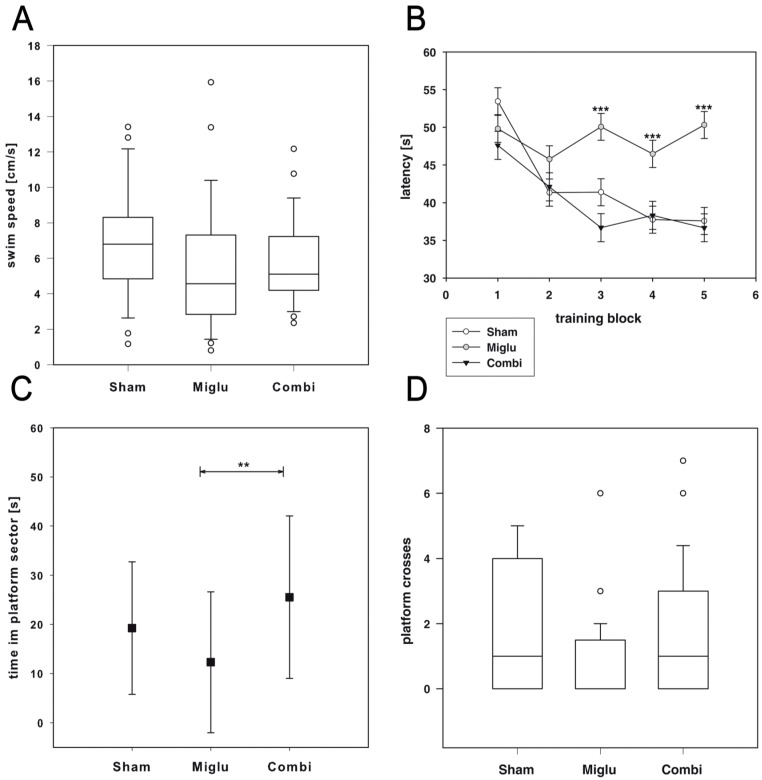
Water maze test. (**A**) All treatment groups (*n*_sham_ = 29, *n*_miglu_ = 26, *n*_combi_ = 26) showed similar swim performance (*p* = 0.147); (**B**) escape latencies for the individual trials were averaged by training block. Only the miglu-group shows no improvement of spatial cognitive capabilities. During training blocks 3–5 the miglu-group showed significant worse platform finding latencies than the sham- and combi-groups; (**C**) after platform removal the miglu-treated mice spent significantly less time in the respective sector than combination-treated mice; (**D**) differences in platform crosses did not reach significance. Asterisks indicate significant differences between treatment groups after two-way analysis of variance and Holm–Sidak post hoc analysis (** *p* < 0.01, *** *p* < 0.001). Scatter plot and line chart data are represented as mean ± SEM (**B**) or mean ± SD (**A**,**C**,**D**) Box plots depict the animal groups graphically by displaying the following descriptive statistical parameters: the median, the upper and lower quartiles, and outliers (circles) that lie outside the 10th and 90th percentiles (whiskers).

The Morris Water maze test was used for evaluating effects of treatment on spatial learning capabilities of wild-type mice. Two way repeated measures ANOVA revealed significant differences (F_2, 79_ = 3.746, *p* = 0.028) in latency of finding the hidden platform (Figure 3B). Since there are no differences in swim speed (Figure 3A), these differences must be an effect of the pharmacologic treatment with a statistically significant interaction between treatment group and test block (F_8, 1685_ = 3.917, *p* < 0.001). All pairwise multiple comparison procedures (Holm–Sidak method) revealed significantly increased latencies for miglustat-treated mice from blocks 3 to 5 compared to sham- and combination-treated mice (*p* ≤ 0.001).

After removal of the platform we observed significant different times the animals spent in the platform sector (F_2, 79_ = 5.61, *p* = 0.005, Figure 3C). Post hoc test (Holm–Sidak) revealed that miglustat-treated mice spent significantly less time in the platform sector than combination-treated mice (*p* ≤ 0.001). We also detected significant differences in numbers of platform crossings (ANOVA on Ranks, H_2_ = 6.535, *p* = 0.038 Figure 3D). However, all pairwise multiple comparison procedures (Dunn’s method) did not reveal significant differences between the groups.

### 2.5. Enhanced Anxiety by Miglustat and Combination Treatment

**Figure 4:**
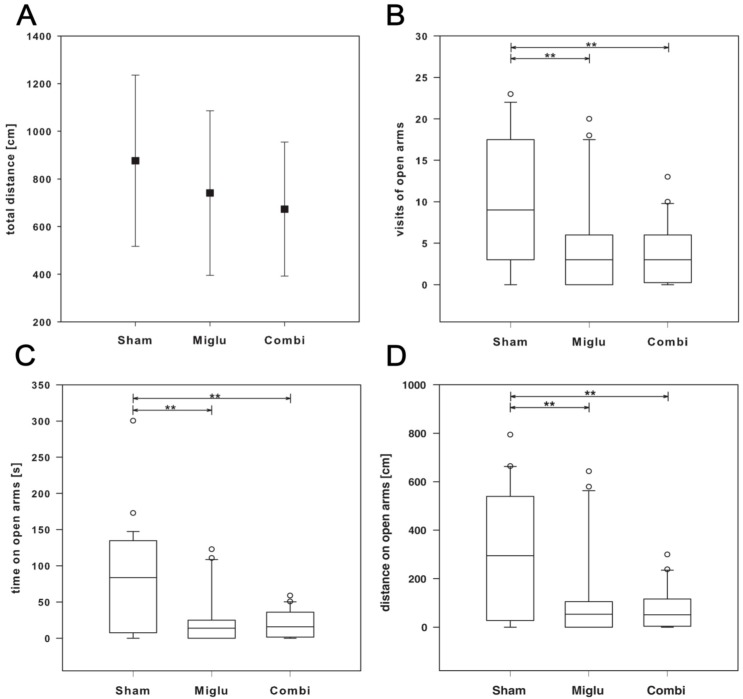
Elevated plus maze test (EPM). (**A**) Motor activity (*n*_sham_ = 29, *n*_miglu_ = 20, *n*_combi_ = 20) was not altered in treated mice as shown by total walking distance, which did not differ significantly between groups (*p* = 0.103); (**B**) number of visits of open arms; (**C**) distance on open arms; (**D**) time on open arms. Asterisks indicate significant differences compared to the sham group (** *p* < 0.01). Data of scatter plot are represented as mean ± SD. Box plots depict the groups graphically by displaying the following descriptive statistical parameters: the median, the upper and lower quartiles, and outliers (circles) that lie outside the 10th and 90th percentiles (whiskers).

The elevated plus maze test (EPM) was done at P56 to investigate the effect of the drug administration on anxiety. There is not a statistically significant difference (*p* = 0.103) in walking speed, all treatment groups display comparable motor activities (Figure 4A).

Analysis of the number of visits of open arms using one way ANOVA on ranks (Kruskal–Wallis) revealed a statistically significant difference (H_2_ = 10.012, *p* = 0.007). All pairwise multiple comparison procedure (Dunn’s method) showed statistical differences (*p* < 0.05) between sham- and both treatment-groups. Both miglu- and combi-groups visited the open arms of the elevated plus maze significantly less frequent indicating enhanced anxiety compared to the sham-group (Figure 4B). This result is confirmed by analyzing the walking distance on open arms. One way analysis of variance on ranks revealed statistically significant differences (H_2_ = 10.678, *p* = 0.005). All multiple comparison procedures (Dunn’s method) confirmed the statistical differences (*p* < 0.05) between sham- and miglu-groups and between sham- and combi-groups (Figure 4D). Analyzing the time on open arms revealed similar results. ANOVA on ranks (Kruskal–Wallis) confirmed this finding (H_2_ = 11.412, *p* = 0.003) and multiple comparison procedure revealed statistical differences (*p* < 0.05) between sham- and miglustat- and sham- and combination treatment (Figure 4C). Thus, the analysis of three parameters of the elevated plus maze support the enhanced anxiety of both treatment-groups compared to the sham-group.

### 2.6. Neither Treatment Influenced Explorative Behavior in Open Field Test

**Figure 5:**
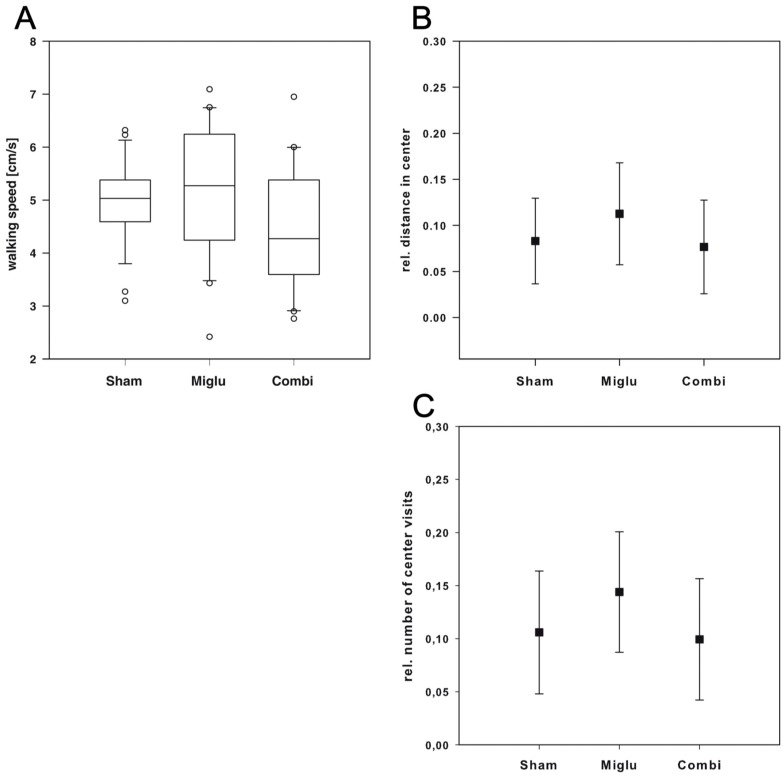
Open Field test. (**A**) The general motor activity was similar among groups (*n*_sham_ = 29, *n*_miglu_ = 20, *n*_combi_ = 20); (**B**) there are no significant differences between groups (*p* = 0.057) in the relative center distance; (**C**) the relative frequency of center visits revealed significant differences between the treatment groups (*p* = 0.032), but post hoc test (Holm–Sidak) failed to show significant differences between different groups. Scatter plots are represented as mean ± SD. Box plots depict the groups graphically by displaying the following descriptive statistical parameters: the median, the upper and lower quartiles, and outliers (circles) that lie outside the 10th and 90th percentiles (whiskers).

The Open Field test was conducted to assess explorative locomotor activity and anxiety. Like in all other tests, there was no difference in walking speed (Figure 5A) between all three groups (H_2_ = 5.605, *p* = 0.061). By analyzing the ratio of center distance to total distance (Figure 5B) we observed no significant differences between the three treatment groups (F_2, 66_ = 2.99, *p* = 0.057). In addition, we observed no significant differences between all three groups by analysis of the ratio of center to total visits (Figure 5C). One way ANOVA revealed the existence of significant differences (F_2, 66_ = 3.614, *p* = 0.032), but pairwise multiple comparison procedures (Holm–Sidak method) revealed no significant differences between particular groups.

### 2.7. Miglustat Treatment and Combination Treatment Differed in Pain Sensitivity

**Figure 6:**
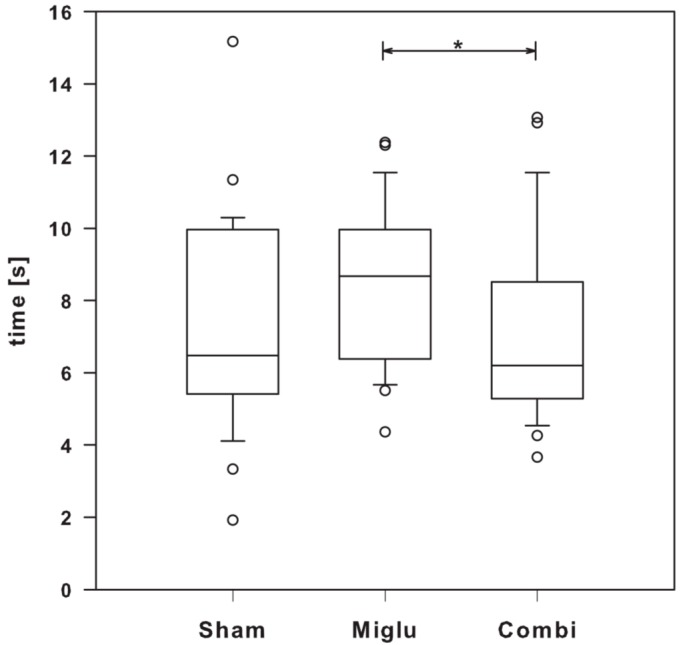
Hot-plate test. Significant differences in pain sensitivity were seen between miglu- and combi-groups (*n*_sham_ = 29, *n*_miglu_ = 26, *n*_combi_ = 26). However, none of the treatment groups was significant different from the sham-group. Asterisks indicate significant differences between treatment groups after one way analysis of variance on ranks and Dunn’s all pairwise multiple comparison procedures (* *p* < 0.05 ). Box plot depicts the animal groups graphically by displaying the following descriptive statistical parameters: the median, the upper and lower quartiles, and outliers (circles) that lie outside the 10th and 90th percentiles (whiskers).

For analysis of the effect of therapy on pain sensitivity the hot-plate test was used. Despite one way analysis of variance on ranks revealed significant differences (H_2_ = 6.142, *p* = 0.046) a multiple comparison procedure (Dunn’s Method) revealed only minor significant differences between miglu- and combi-groups, but none of these treatment groups were significantly different from the sham-treated mice (Figure 6).

## 3. Discussion

The main outcome of the present study is that substances used for the treatment of NPC1 disease affect the postnatal development of BALB/c wild-type mice and their adult behavior. Miglustat alone or in combination with allopregnanolone/cyclodextrin have proven substantial positive therapeutic effects in *Npc1*^−/−^ mouse mutants [12,13] and humans [6,7,19,20,21]. The benefit is explained by the interference of the drugs with the lysosomal cholesterol traffic that is altered due to the defective NPC1 protein [5,22].

In *Npc1*^−/−^ mice the drugs reduced concentrations of cholesterol and other lipids including the glycosphingolipids GM2 and GM3 in brain, liver, and other organs [12,16,23,24]. We here hypothesize that by using the respective drugs in wild-type mice, concentrations of cholesterol and other lipids were decreased below normal level, interfering with normal cellular or membrane functions. Interestingly, Davidson and others showed that therapeutically efficacious doses of HPβCD in wild-type mice and also cats led to ototoxicity, possibly by lipid depletion of the delicate hair cells [16,17,18]. Moreover, in *Npc1*^−/−^ CHO cells Tanaka et al. [25] found a high tolerability against HPβCD toxicity compared with wild-type CHO cells and discussed this phenomenon with respect to cholesterol-solubilizing activity of cyclodextrin without giving an explanation for the precise molecular mechanism. Kondo et al. [26] pointed out that the cholesterol-solubilizing ability of HPβCD attenuated effects against NPC abnormalities and on the other hand induced cytotoxicity, and Frank et al. [27] described that methyl-β-cyclodextrin (MβCD) mediated cholesterol depleted hippocampal neurons exhibit an impaired NMDA receptors-mediated synaptic plasticity in rat hippocampus. A recent study indicates that MβCD can affect even both presynaptic and postsynaptic properties, and that some effects of MβCD are unrelated to cholesterol chelation. In crayfish extensor muscle fibers MβCD dramatically reduced responses to local application of l-glutamate by iontophoresis, suggesting a direct effect on glutamate receptor function [28].

Miglustat is an inhibitor of gylcosylceramide-synthetase and by this used as substrate reduction therapy [8,29]. A systematic review on later findings of clinical trials [21] revealed that miglustat can slow down the progression of neurologic symptoms in all NPC patients, yet the therapeutic benefit is greater in those with a late diagnosis compared with early childhood onset. Although miglustat provided the proof-of-principle for the efficacy of substrate reduction strategies, miglustat still has its limitations, which are mostly related to unwanted side effects including visceromegaly, hematologic abnormalities, diarrhea, intestinal carbohydrate malabsorption and weight loss [6,10,30,31,32,33]. However, our treated mice did not show any obvious gastrointestinal symptoms.

A recent study on BALB/c *Npc1*^−/−^ mice revealed that the progressive neurologic symptoms are accompanied by an impairment of both induction and maintenance of long term synaptic potentiation associated with the lack of ERKs phosphorylation [34]. This lack of synaptic plasticity was restored by miglustat administration to normal levels. The authors discuss that the cholesterol dysmetabolism in NPC1 may be responsible for the observed synaptic plasticity phenomena impairment and that miglustat corrects the abnormal lipid trafficking without having a direct effect on cholesterol metabolism. However, miglustat administered to wild-type mice possibly can induce changes in the plasma membrane cholesterol content and in the glycosphingolipids/cholesterol ratio. In particular, it may affect lipid rafts, cell membrane microdomains where many transductive signaling processes are generated, i.e., important regulators in the neurotransmitter release process and of glutamate receptor activity [35,36].

Despite these numerous studies on NPC1 patients and BALB/c *Npc1*^−/−^ mutant mice, only little is known about the effects of livelong treatment with miglustat alone or in combination with allopregnanolone/cyclodextrin starting at P7 in healthy BALB/c mice.

We observed significantly reduced body weights in wild-type mice due to combination treatment but not due to miglustat treatment. At day of sacrifice the combination-treated mice body weights were significantly lower than those of sham- and miglu-groups. Therefore, the reduced body weight is very likely an effect of cyclodextrin. Whereas for allopregnanolone no side effects are known, cyclodextrin has been reported to cause body weight loss due to binding to blood lipids [37,38]. These particular studies used α-cyclodextrin, whereas we used 2-hydroxypropyl-β-cyclodextrin (HPβCD). However, the ability to form complexes with hydrophobic compounds is a central feature of all cyclodextrins. Substantial interaction with several relevant lipids was recently demonstrated for HPβCD and other cyclodextrins [16]. It was also shown that methyl-β-cyclodextrin (MβCD) perturbs formation of clathrin-coated endocytotic vesicles through cholesterol depletion [39] and induces vesicle disruption and solubilization through affinity for both lipid components of liposomes, cholesterol and phosphatidylcholine [40,41].

Whereas the body weights of sham- and miglu-groups were not distinguishable, the brains of miglu- and combi-groups were significantly smaller than those of sham-treated mice. The smaller brains of the combi-group could be a consequence of the reduced body weight, but this cannot be the explanation for the lighter brains of the miglu-group. Possibly a reduced membrane biosynthesis or reduced lipid storage due to inhibition of the glucosylceramide biosynthetic pathway by miglustat is responsible for the reduced brain weights of combination- and miglustat-treated mice [42]. For methyl-β-cyclodextrin (MβCD) it has recently been shown that plasma membrane cholesterol depletion affects early forebrain patterning in Xenopus [43]. MβCD-injected embryos displayed reduced eyes or lack eyes entirely, most likely because of loss of neural tissue from where these structures derive. Additionally, it can be speculated that miglustat- and cyclodextrin-mediated changes in lipid pattern can interfere with myelination, whose essential parts start at about P10 in rodents [44,45], i.e., after the induction of our treatment regime.

We observed no differences in motor capabilities of miglu- and combination-treated mice compared with sham-treated animals at both P35 and P60 in the accelerod test and all other tests. There are no pharmacologic effects on motor performance in the accelerod test, neither in walking and swimming speeds in the elevated plus maze test, the open field test and the water maze test. We conclude that none of the used drugs has any effect on the motor systems of wild-type mice. This is in accordance with our previous study that revealed no motor differences between combination-treated and sham-treated control mice [13]. Since accelerod performance of presymptomatic combination-treated mutant mice was significantly better than combination-treated and sham-treated mutant *Npc1* mice, we speculated a pronounced motor performance effect of the pharmacologic treatment. However, this study clearly disproves this speculation in wild-type mice.

The Morris water maze test revealed impaired spatial learning capabilities in wild-type mice treated only with miglustat, whereas combination-treated mice performed not differently from sham-treated mice. After removal of the hidden platform we observed the same differences in time spent in platform sector and the number of platform crosses. However, post hoc test failed to isolate the groups with statistically significant differences in number of platform crosses. Water maze performance is influenced by sex, age, nutrition, stress and the background strain (reviewed in [46]). Some strains failed to show a quadrant preference during the probe trial, despite improvement during acquisition training [47,48]. Van Dam et al. [48] demonstrated that BALB/c mice never displayed a clear preference for the target quadrant. The authors conclude that BALB/c mice were unable to adequately learn and remember the position of the escape platform. For our wild-type mice with a BALB/c strain background we can only partly confirm this statement, since the sham-treated mice clearly learned the task (Figure 3B). However, a considerable inter-individual variability was observed regarding the platform crossings (Figure 3D). Combination-treated mice also learned the task as well as the sham-treated ones, whereas the miglustat-treated mice performed significantly worse. Interestingly, a common adverse reaction of miglustat treatment is cognitive dysfunction [49]. It can be speculated that this negative effect of miglustat is compensated by cyclodextrin in the combination treatment—the molecular basis being still unclear. However, it was recently demonstrated that distribution of agents in the brain, e.g., adenylate cyclase activating polypeptide, can be dramatically altered by cyclodextrins, whereby different cyclodextrins produced specific distribution patterns [50]. It can be speculated, that cyclodextrin also altered the distribution pattern of miglustat in the hippocampus that is essential for spatial learning and memory in rodents [51].

The elevated plus maze (EPM) test is a commonly used test for detecting psychiatric symptoms in rodents [52,53,54]. Our tests revealed significantly enhanced anxiety of both, the miglu- and combi-groups compared to the sham-group. This is seemingly in line with known psychiatric side effects of miglustat-induced depression [49].

Locomotor activity and anxiety can be evaluated by placing the mouse in a square open field arena (OF) under standard room lighting [55]. This paradigm mimics the natural conflict in mice between the tendency to explore a novel environment and the tendency to avoid a brightly lit open area [56,57,58,59]. Like in the EPM paradigm we did not detect any differences in explorative behavior. However, unlike in the EPM we found no differences in ratio of center distance to total distance that can be taken as a measure of anxiety [60]. Whereas the elevated plus-maze is a considerably good test and widely used for anxiety behavior, the OF test is a good measure of locomotive and exploratory behavior in rodents. Interestingly, others reported that there were no significant correlations between the important anxiety parameters evaluated in EPM and OF [61,62].

The hot-plate test is one of the most commonly used tests for determining the analgetic efficacy of experimental drugs in rodents [63]. We detected significantly reduced pain sensitivity in the miglu-group compared to the combi-group, but both treatment groups were not significantly different from the sham-group. A serious adverse reaction reported with Zavesca^®^ treatment in clinical studies was peripheral neuropathy [64] and reduced sensation to touch [65]. It can be speculated that the miglustat-induced reduction of pain sensitivity was compensated by cyclodextrin in the combination treatment.

Although miglustat or combination treatment had positive effects on mutant *Npc1* mice [12,13,14,16], wild-type mice did not profit from the drug administration, since none of the drug-treated groups performed better than sham-treaded mice in any of the behavioral tests. In contrast, miglustat- and combination treatment caused side effects on physiological, psychiatric or cognitive traits. Combination treatment with miglustat and additional administration of HPβCD and allopregnanolone caused body weight loss, whereas the weights of mice treated with miglustat alone were indistinguishable from their sham-treaded littermates. The effects of the drugs on motor capabilities were not significant. Treatment with miglustat impaired spatial learning capabilities and reduced pain sensitivity slightly but significantly. Both effects of miglustat were ameliorated to sham treatment levels by combined administration of miglustat with HPβCD and allopregnanolone. In conclusion, additional administration of HPβCD and allopregnanolone ameliorated most but not all side effects of miglustat. It can be speculated, however, that HPβCD and not allopregnanolone acted beneficially as clinical trials only with cyclodextrin and experimental data ascribed cyclodextrin a high therapeutic potential in NPC disease [12,16,24,66].

To strengthen our findings more experiments are needed including several groups with monotherapies. While the allopregnanolone’s beneficial effect is supposed to be synergistic [12], a worthwhile approach would be to determine which of the monotreatments with miglustat or cyclodextrin contributes to disease deceleration in *Npc1*^−/−^ mice and which of them caused side effects in *Npc1*^+/+^ mice described in this study.

## 4. Methods

### 4.1. Animals

Wild-type BALB/c breeding pairs were obtained from Charles River (Charles River Laboratories, Sulzfeld, Germany). Offspring were housed in groups of 2–5 with free access to food and water. A 12-h light–dark cycle was maintained (light on from 6.00 a.m. to 6.00 p.m.) with a temperature of 22 °C and a relative humidity of about 50%–60%. Altogether, 81 male wild-type mice were involved in this study. Mice were divided into three groups: (i) Sham-treated group (sham-group, *n* = 29, injected with the respective amounts of 0.9% NaCl (*n* = 15) or pure needle penetrations without volume injection (*n* = 14) according to the treatment plan of the combination-treated group); (ii) combination-treated group (combi-group, *n* = 26, combined β-cyclodextrin/allopregnanolone/miglustat injections) and (iii) miglustat-treated group (miglu-group, *n* = 26, treatment only with miglustat). As the NaCl injected sham-group and the volumeless injected sham-group did not differ in the behavioral tests, we summarized them to one sham-group. Volumeless injections were done in order to rule out that respective nociceptive stimuli alone can cause behavioral changes. All animal procedures used in the experiments were approved by the local Animal Use and Care Committee of Mecklenburg-Western Pomerania (approval ID: 7221.3-1.1-088/10). All institutional guidelines for animal welfare and experimental conduct were followed. All efforts were made to minimize suffering.

### 4.2. Pharmacologic Treatment

Starting at postnatal day 7 (P7) and thenceforth, mice of the combi-group were injected weekly with 2-hydroxypropyl-β-cyclodextrin/allopregnanolone (25 mg/kg allopregnanolone dissolved in 40% 2-hydroxypropyl-β-cyclodextrin in Ringer’s solution, 4000 mg/kg, i.p., all from Sigma-Aldrich, Munich, Germany). Additionally, these mice were daily injected with miglustat, dissolved in 0.9% NaCl solution, 300 mg/kg i.p. (*N*-butyldeoxynojirimycin, Zavesca; Actelion Pharmaceuticals, San Francisco, CA, USA) from P10 to P23. From P23 onwards until termination of experiments mice were fed standard chow with embedded miglustat resulting in daily intake of 1200 mg/kg miglustat. The miglu-group was treated like the combi-group, but without administration of cyclodextrin/allopregnanolone, instead mice got vehicle. Mice of the sham-group were injected like those of the combi-group at the various time points with the respective volumes of 0.9% NaCl or without volume and were fed with chaw without drugs.

### 4.3. Behavioral Testing

**Figure 7:**
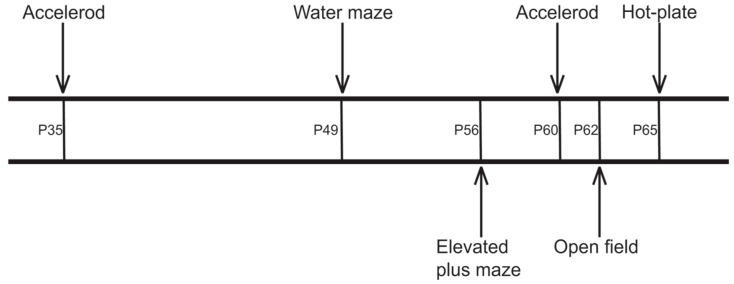
Time points of behavioral tests. P—postnatal day.

A battery of behavioral tests was accomplished at different time points (Figure 7). Tests without a training phase (elevated plus maze, open field, hot-plate) were performed in the dark phase of the light-dark cycle. The experimenter was blinded to the treatment the mice received. The sequence of the tests was chosen for starting with training intensive tests including handling, followed by the anxiety related tasks without further handling and novelty stress. In this line the hot plate test was put to the end [67,68,69].

#### 4.3.1. Accelerod Test

The rotarod/accelerod test is widely used for evaluating motor coordination, balance and ataxia [70]. The accelerod test has been shown to be more sensitive than the rotarod test in detecting motor function deficits and in providing more consistent results [71]. To determine balance and motor coordination an accelerod system (TSE Systems, Bad Homburg, Germany) for mice was used on P35 and P60. The apparatus consisted of a base platform and a rotating rod (3 cm diameter, 11.4 cm width) with a non-skid surface for training each mouse got 4 test trials per day at constant 12 rpm for 2 min on 2 consecutive days. When operated in the acceleration modus, the rotation increased from 4 to 40 rpm in 30 s steps within 5 min. Each mouse got 4 trials per day on 2 consecutive days. During training the latency of first fall down and the number of fall downs during the training trials were recorded. During accelerod test trials the latency and reached rpm were recorded.

#### 4.3.2. Watermaze Test

This test developed by Richard Morris was used to assess spatial reference and working memory [51]. The water maze consisted of a black circular plastic tank, 102 cm in diameter and 50 cm deep. It was filled with fresh water on each test day with a temperature of 19 °C. A black painted target platform (diameter 11 cm, height 18.5 cm) was submerged 1.5 cm below the water surface and the pool was surrounded by different spatial cues mounted on the walls of the room (extra-maze cues). During training the mice first accustomed to locate the submerged platform under dim light conditions (indirect illumination, 3.5 Lux). On the first day mice were allowed to swim in the pool for 60 s to become acquainted with the test apparatus. Twenty-four hours later animals were trained within two blocks consisting of four trials for a total of three days. Starting points varied for each mouse, whereas the location of the platform remained constant throughout the whole training period. The mice were allowed 60 s to find the platform. Animals that did not find the platform in 60 s were gently guided to it. All animals got 30 s rest period on the escape platform between trials. The escape latency was recorded by the VideoMot2 Software (TSE Systems, Bad Homburg, Germany). The probe trial was performed after the last training block within 60 s during which the platform was removed from the pool. The starting position of probe trial was located opposite to the quadrant which originally contained the platform. The frequency the mice crossed the former platform position (platform crossings) and time spent in the respective sector were calculated by the VideoMot2 tracking software (TSE Systems, Bad Homburg, Germany).

#### 4.3.3. Elevated Plus Maze Test

The elevated plus maze test is probably the most popular of all currently available animal models of anxiety [52] and was conducted to assess anxiety-related behavior at P56, using a custom made apparatus consisting of two open arms and two closed arms positioned at 90° angles (arm length 425 mm, arm width 145 mm, wall height 225 mm, width of ledges 10 mm). The arms were arranged in a way that two pairs of identical arms were placed opposite to each other. Arms emerged from a central platform and the entire apparatus was elevated 50 cm above the floor. The test was performed under dim light conditions (3.5 Lux). The mice were kept 1 h before test start at dimmed light in the test room in order to become familiar with the novel condition. Each mouse was placed at the center of the maze facing an open arm, and the number of entries and the time spent in closed and open arms were recorded during a 10 min observation period by a video tracking system (VideoMot2, TSE Systems, Bad Homburg, Germany). After each test the maze arms were wiped clean by using a wet towel. Anxiety was measured by the number of visits, distance and time spent in open/closed arms. The general motor activity was evaluated through the total distance and total visits.

#### 4.3.4. Open Field Test

Open field test was originally described by Hall in 1934 [72] and was conducted to assess exploratory locomotor activity and emotionality in a 15 min trial during the mice are subjected to a novel environment from which escape is prevented by surrounding walls. One hour before starting the test, the animals were kept at dimmed light in the examination room to become familiar with the novel condition. For the test, the mice were placed in a novel environment inside of an isolation box (TSE-Systems, Bad Homburg, Germany) with a square open field arena of 50 cm × 50 cm. The floor was divided by tracking-software into 16 equal squares by black-colored grids. For analysis, the chamber was virtually divided into central and peripheral zones. Mice were not previously habituated to the locomotor activity chamber. Illumination of the open field was provided by a white photo bulb providing 100 Lux. Environmental odors were removed by thorough cleaning the open field after each session to avoid influences of the behavior by odor trials. The movements were recorded by a video camera placed inside the isolation box and tracked using the VideoMot2 Software (TSE Systems, Bad Homburg, Germany). The distance traveled and time spent by the mice in either the center or periphery of the open field was analyzed.

#### 4.3.5. Hot-Plate Test

The hot-plate test is a widely used test of the pain response in animals proposed by Eddy and Leimbach in 1953 [73]. One hour after begin of the dark phase the mouse was placed on the surface of a hot-plate adjusted to 52.5 °C. A transparent glass cylinder was used to keep the animal on the heated surface of the plate. The time of latency is defined as the time period between the zero point, when the animal is placed on the hot-plate surface, and the time when the animal licks its paw or jumps off to avoid thermal pain. The time of maximum permanence permitted on the hot surface was 15 s.

### 4.4. Statistics

Data were subjected to one- or two-way ANOVA with one between-subject factor (application) and with repeated measurements depending on the data structure. The Holm–Sidak approach was used for adjustment for multiple testing for post hoc comparisons. A critical value for significance of *p* ≤ 0.05 was used throughout the study. In case of non-normally distributed data, data were subjected to Kruskal–Wallis one- or two-way ANOVA on ranks and displayed as box plot. Dunn’s test was used for post hoc comparisons after ANOVA on ranks to adjust for multiple testing. Adjustment for multiple testing was therefore only done for post hoc comparisons separately for different ANOVAs and does not ensure that type I error is 5% or less with regard to the whole study.
